# Field testing of recombinant subunit vaccines against *Teladorsagia circumcincta* in lambing ewes demonstrates a lack of efficacy in the face of a multi-species parasite challenge

**DOI:** 10.3389/fpara.2024.1360029

**Published:** 2024-03-25

**Authors:** Alasdair J. Nisbet, Tom N. McNeilly, Daniel R. G. Price, Yvonne Bartley, Margaret Oliver, Dave McBean, Leigh Andrews, Gillian Mitchell, Rachael Duncan, Sarah Brocklehurst, Fiona Kenyon

**Affiliations:** ^1^ Vaccines and Diagnostics Department, Moredun Research Institute, Edinburgh, United Kingdom; ^2^ Disease Control Department, Moredun Research Institute, Edinburgh, United Kingdom; ^3^ Biomathematics & Statistics Scotland, Edinburgh, United Kingdom

**Keywords:** parasitic gastroenteritis, nematode vaccine, teladorsagiasis, field trial, recombinant

## Abstract

**Introduction:**

We previously demonstrated efficacy of an 8-antigen recombinant subunit vaccine against a single species homologous *Teladorsagia circumcincta* challenge in lambs and in lambing ewes in pen trials. We subsequently demonstrated efficacy of a simplified, 2-antigen, version of this vaccine in lambs in pen trials. Here, we test both vaccines in lambing ewes in a field setting.

**Methods:**

In the work presented here, 12 adjacent plots were seeded with a mixed infection of several common species of parasitic nematodes of sheep in temperate regions, including *T. circumcincta*. Ewes (n = 144), in groups of 12, grazed for 2 years on these plots and, in the first year, six of these groups of ewes were vaccinated with a 2-antigen prototype vaccine against *T. circumcincta* prior to mating and then again prior to lambing. In the following year these ewes were immunised again, this time with the 8-antigen prototype vaccine against *T. circumcincta* prior to mating and then prior to lambing. Throughout both seasons antigen-specific serum antibody levels in ewes and faecal worm egg counts (FEC) in ewes and their lambs were monitored, along with nematode species diversity at lambing.

**Results:**

Immunised ewes produced elevated serum antibody levels to each of the vaccine antigens following immunisation but their FEC levels were not statistically significantly impacted by vaccination with either vaccine. FEC levels were also not impacted in lambs co-grazing the pastures with these immunised ewes. Nematode species diversity was not significantly impacted by vaccination in either year.

**Discussion:**

The immunosuppressive effects of co-infecting gastrointestinal nematodes, the absence of vaccine cross-protection against co-infecting species and the influence of the periparturient relaxation in immunity probably all contributed to the inability of either vaccine to protect against *T. circumcincta* infection in field trials in the work presented here.

## 1 Introduction

Vaccination is emerging as a possible strategy to address the gap in available control methods for gastrointestinal nematodes (GIN) caused by the development of resistance against currently-available anthelmintics (Britton et al., 2020). However, the only commercially available vaccine for this purpose currently is Barbervax™, which is specific for *Haemonchus contortus* and is limited in its commercial scope because it consists of native gut glycoproteins extracted from the adult parasites (Nisbet et al., 2016a). Recently, progress has been made to develop a recombinant subunit vaccine to control *Teladorsagia circumcincta*, the primary cause of parasitic gastroenteritis (PGE) in small ruminants in temperate regions worldwide (Nisbet et al., 2013). In initial studies, an 8-antigen recombinant subunit prototype vaccine reduced mean cumulative faecal egg counts (cFEC) from *T. circumcincta*-challenged 6-month old lambs by 70% and 58%, respectively, over a 6 or 10-week period after challenge (Nisbet et al., 2013). Vaccinated lambs also had up to 75% lower mean adult nematode burdens than adjuvant-only controls (Nisbet et al., 2013). In lambing ewes, that displayed a periparturient relaxation in immunity (PPRI), vaccination with this complex prototype vaccine resulted in a 45% reduction in mean cFEC compared to control animals which had received adjuvant only (Nisbet et al., 2016b). A simplified version of the vaccine was designed, based on relationships between antigen-specific antibody levels, avidity measurements and parasitological parameters of efficacy analysed for each of the eight proteins in the previous trials, and this led to the testing of a vaccine comprising two of the original eight antigens; a non-enzymically active (“mutated”) version of *T. circumcincta* apyrase-1 (mTci-APY-1) and *T. circumcincta* metalloproteinase-1 (Tci-MEP-1) (Nisbet et al., 2019). In a series of trials of the original eight-antigen vaccine, the strongest correlations between percentage reduction in cFEC and avidity were obtained for Tci-APY-1 in relation to total antigen-specific IgG in sera. Serum and abomasal mucosal IgG and IgA of control (parasite challenged) lambs strongly recognised Tci-APY-1 and Tci-MEP-1 indicating that these two antigens are most effectively recognised by the parasite-induced antibody response developing during exposure. Mean cFEC levels in 6-month old lambs vaccinated with this 2-antigen recombinant subunit prototype vaccine and challenged with *T. circumcincta* were reduced by 43% compared to the controls (Nisbet et al., 2019).

An ideal scenario for the use of such vaccines in practice would be to use them in pregnant ewes to reduce pasture contamination during the PPRI, thus reducing exposure of young, immunologically naive lambs to high levels of infection as they start to graze. As a second line of defence, these lambs could also be vaccinated once they reach immunological competence to respond to the vaccine. In relation to this, the original prototype vaccine against *T. circumcincta* was effective in 3-month-old lambs (Liu et al., 2022). The work presented here builds towards that scenario by testing the efficacy of the simplified and the complex anti-*T. circumcincta* vaccines in lambing ewes grazing a pasture contaminated with a mixture of GIN species typically found in temperate sheep production systems.

## 2 Results/discussion

### 2.1 Impacts of vaccination on parasitism of ewes and contamination of pasture

**Figure 1:**
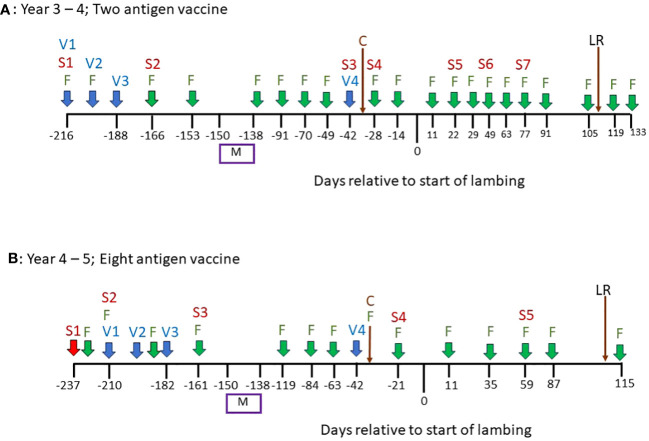
An overview of the vaccination and sampling schedules in a field experiment to measure the impacts of vaccination on gastrointestinal parasitism in pregnant sheep. **(A)** Timeline of events in Years 3–4 of the study. **(B)** Timeline of events in Years 4–5 of the study. “V” = date of vaccination; “S” = date of serum sampling; “F” = date of faecal egg counts on all animals in trial; “M” = mating period; “C” = Clostridial vaccine, all animals in trial; “LR” = lambs removed.

**Figure 2:**
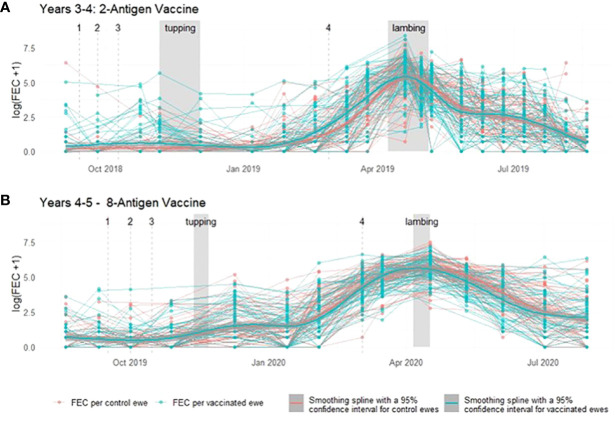
Faecal egg count data (total strongyles) from ewes in two field trials of a *T. circumcincta* recombinant subunit vaccine. **(A)** Log transformed data from each ewe vaccinated with a 2-antigen sub-unit vaccine in Years 3–4 of the study (blue lines) or unvaccinated “Control” ewes (red lines). **(B)** Log transformed data from each ewe vaccinated with an 8-antigen sub-unit vaccine in Years 4–5 of the study (blue lines) or unvaccinated “Control” ewes (red lines). Dates of mating (“tupping”) and dates of lambing are shown in shaded grey. Dates of vaccine doses are indicated by a dashed grey line and labelled 1–4. Cubic regression smoothing splines with 95% confidence intervals were fitted to the data points for the vaccinated and for the unvaccinated ewes.

**Figure 3:**
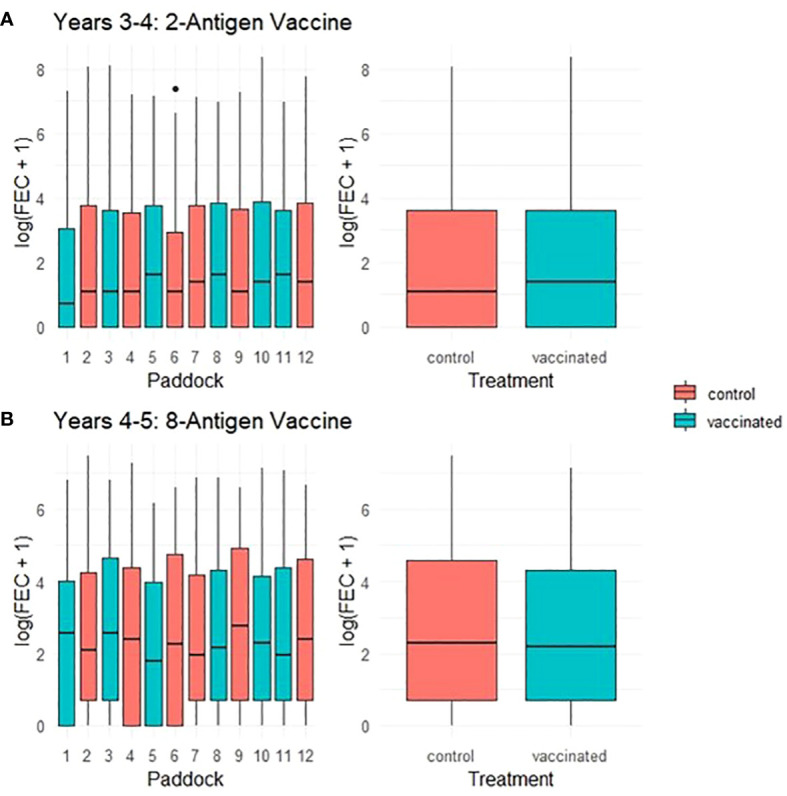
Box plots of faecal egg count data (total strongyles) over all sampling times from ewes in two field trials of a *T. circumcincta* recombinant subunit vaccine. **(A)** Log transformed data for ewes vaccinated with a 2-antigen sub-unit vaccine in Years 3–4 of the study (blue) or unvaccinated “Control” ewes (red). **(B)** Log transformed data for ewes vaccinated with an 8-antigen sub-unit vaccine in Years 4–5 of the study (blue) or unvaccinated “Control” ewes (red). The box plots on the left side of each panel show logged FECs for each paddock while those on the right side show logged FECs for the vaccinated or control groups as a whole.

Throughout a 2 year period, during which ewes were vaccinated with *T. circumcincta* recombinant subunit vaccines in each year and exposed to nematode-contaminated pastures, the principal measure of vaccine efficacy was impact on faecal egg count (FEC), which was measured regularly throughout the study (**Figure 1**). Neither vaccine demonstrated statistically significant impacts on FEC in either year of the trial (*p* = 0.34 and 0.31 respectively), with FEC for both vaccinated and control groups following similar trends through time, peaking at lambing, irrespective of which vaccine was used (**Figures 2**, **3**). Median FEC data, with interquartile ranges are shown in **Supplementary File 1**. In brief, in Years 3–4 the median FECs (expressed as eggs per gram of faeces) in vaccinated ewes were: 0, 374, and 1 at the point of first vaccination (V1), peak FEC and post-lamb removal, respectively. At the same timepoints in control ewes the median FECs were 0, 369 and 1. In Years 4–5 the median FECs in vaccinated ewes were: 1, 279, and 8 at the point of first vaccination (V1), peak FEC and post-lamb removal, respectively. At the same timepoints in control ewes the median FECs were 1, 324 and 5.

### 2.2 Impacts on parasitism of lambs following vaccination of their parent ewes

**Figure 4:**
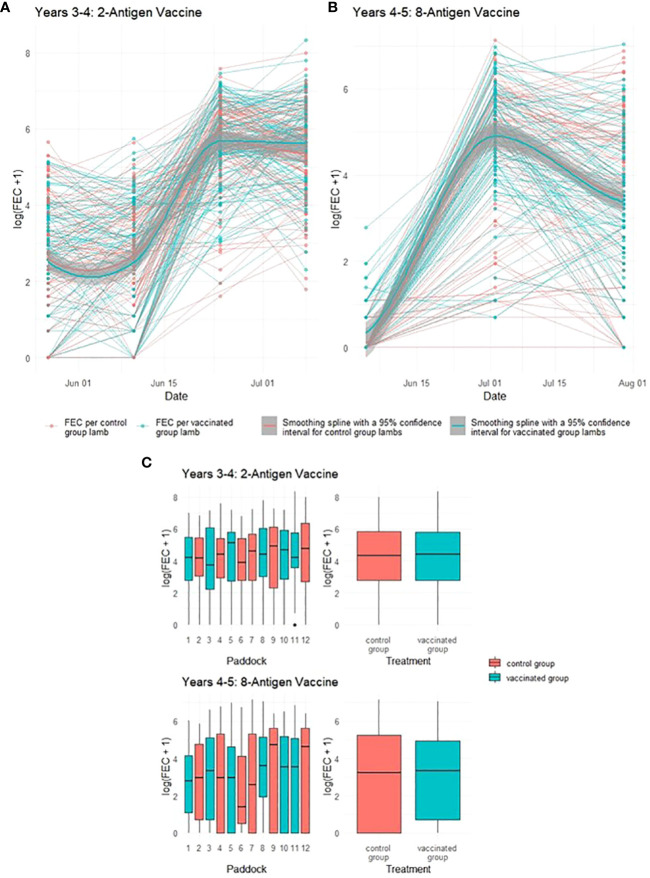
Faecal egg count data (total strongyles) from lambs in two field trials of a *T. circumcincta* recombinant subunit vaccine. **(A)** Log transformed data from each lamb co-grazed with ewes vaccinated with a 2-antigen sub-unit vaccine in Years 3–4 of the study (blue lines) or unvaccinated “Control” ewes (red lines). **(B)** Log transformed data from each lamb co-grazed with ewes vaccinated with an 8-antigen sub-unit vaccine in Years 4–5 of the study (blue lines) or unvaccinated “Control” ewes (red lines). Cubic regression smoothing splines with 95% confidence intervals were fitted to the data points for the vaccinated and for the unvaccinated ewes. **(C)** Box plots of faecal egg count data (total strongyles) over all sampling times from these lambs. The box plots on the left side of **(C)** show logged FEC for each paddock while those on the right side show logged FECs for the vaccinated or control groups as a whole.

One potential impact of reduced pasture contamination from vaccinated ewes would be a reduction in the levels of infective GIN larvae ingested by their co-grazing lambs, leading to impacts on lamb FEC. Neither vaccine demonstrated statistically significant impacts on lamb FEC in either year of the trial (*p* = 0.94 and 0.98 respectively), with FEC for lambs co-grazing with both vaccinated and control ewes following similar trends through time irrespective of which vaccine was used (**Figure 4**). In Year 4, peak median FEC values for lambs from vaccinated ewes were 324 while those from control ewes were 396. In Year 5, peak median FEC values for lambs from vaccinated ewes were 153 while those from control ewes were 198 (see **Supplementary File 1** for full details).

**Figure 5:**
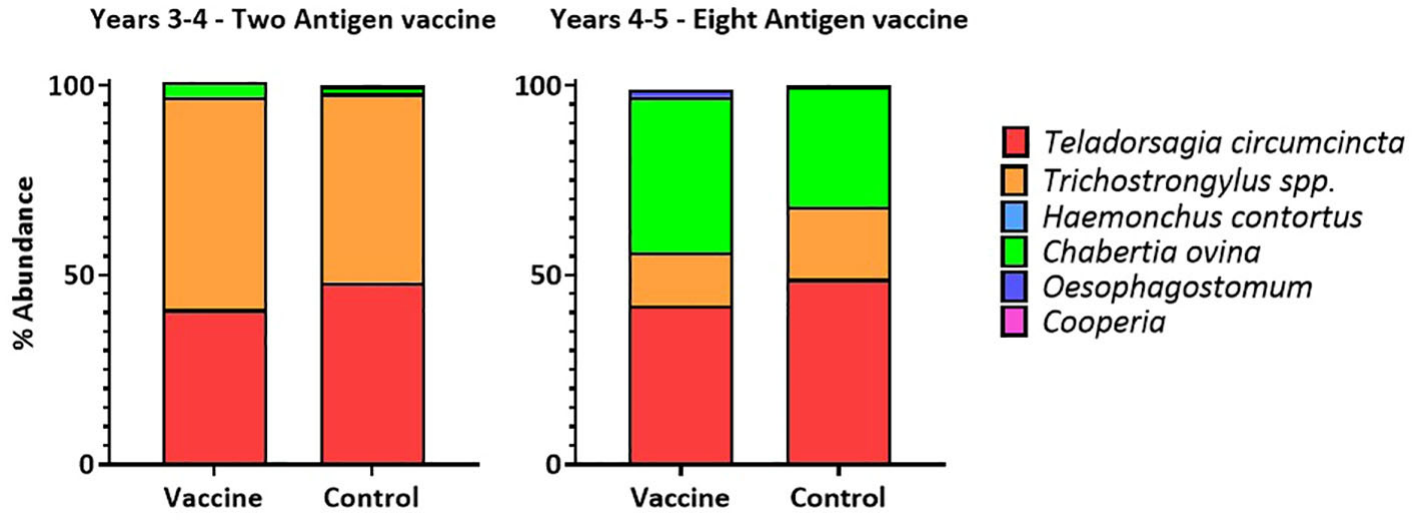
Proportions of six nematode species detected in the faecal egg samples of ewes at lambing in two field trials of a *T. circumcincta* recombinant subunit vaccine. Data shown are mean percentages from 6 paddocks per treatment (vaccine or control) in each year.

In both years, at lambing, the proportions of six different nematode species were determined in the faecal egg samples taken from ewes. In both years, the average proportion of *T. circumcincta* eggs was slightly, but not drastically, reduced in samples from vaccinated ewes taken at a single timepoint, at lambing (**Figure 5**).

### 2.3 Serological response of ewes to vaccination

Two weeks after the final immunisation in a vaccination schedule of 3 immunisations with 2 recombinant antigens (mTci-APY-1 and Tci-MEP-1), two weeks apart, immunised ewes had high antigen-specific IgG levels against both vaccine antigens and elevated IgA levels against Tci-MEP-1 (**Supplementary Figure 1**). These levels waned over the subsequent 126 days, reducing to levels comparable with control, unvaccinated, animals at that point. Following a booster immunisation 6 weeks prior to the estimated lambing date, the antigen-specific antibody levels were restored but antigen-specific IgG levels fell rapidly during the period of late gestation (**Supplementary Figure 1**).

In the second cycle of this trial, when ewes were vaccinated with the 8-recombinant antigen vaccine cocktail, high antigen-specific levels of serum IgG were generated against each antigen, with the exception of Tci-MIF-1 and Tci-ES20 (**Supplementary Figure 2**) as observed in previous, similar trials in lambing ewes (Nisbet et al., 2016b). The levels of antigen-specific IgG following booster vaccination 6 weeks prior to the estimated lambing date were similar to those in previous trials in ewes with this vaccination schedule (Nisbet et al., 2016b). Levels of antigen-specific serum IgA were increased following initial vaccination for all antigens, with the exception of Tci-MEP-1 and Tci-ES20 and were boosted following the fourth vaccination, 6 weeks prior to lambing for most antigens (**Supplementary Figure 3**).

The mechanisms underlying vaccine-induced immunity against gastrointestinal nematodes are complex and may also be breed- and age-dependent, as well as being influenced by pregnancy status (Piedrafita et al., 2010). Analysis of the vaccine-induced protection observed in five independent pen trials of the 8-antigen prototype vaccine in Texel-cross lambs showed that vaccine efficacy was most highly associated with antibody (mucosal and serum IgG) avidity for two of the vaccine antigens; Tci-APY-1 and Tci-MEP-1 (Nisbet et al., 2019; Palarea-Albaladejo et al., 2023). In addition, activation of gene expression in the abomasal lymph nodes, relating to both immunoglobulin production and activation of eosinophils, was related to the vaccine-induced immunity observed in 3-month-old Canaria Hair Breed (CHB) lambs (Pérez-Hernández et al., 2023) and levels of parasite-specific IgA as well as numbers of globular leucocytes, CD45RA+, CD4+ and CD8+ T cells have also been implicated in this protective response in vaccinated 3-month-old CHB lambs (Pérez-Hernández et al., 2022). In 6-month-old Canaria Sheep high levels of antigen-specific IgA, IgG_2_ against Tci-MEP-1, Tci-SAA-1 and Tci-ASP-1 as well as increased globular leucocyte numbers were associated with vaccine-induced protection (Machín et al., 2021). A recent transcriptomic analysis of the abomasal mucosa from 6-month-old Texel-cross lambs immunised with this vaccine also identified early T-helper type-1 immune responses following vaccination, and prior to parasite exposure, as being correlated with protection (Liu et al., 2022), suggesting that cellular immune responses may also be pivotal to protection.

In the work presented here, elevated levels of IgG and IgA were present in pregnant ewes against the two important antigens, Tci-APY-1 and Tci-MEP-1, in both years of the study following pre-lambing booster vaccinations (V4) but no reduction in FEC was observed in either year during the peri-parturient period. This is in contrast to a previous study with this vaccine in lambing ewes of similar age where vaccination resulted in substantial differences in the timing and amplitude of faecal egg counts between vaccinated and control ewes (Nisbet et al., 2016b), resulting in significant reductions in cFEC. The clearest difference between the prior study and the one reported here is in the parasitological challenge and nutritional conditions – in the Nisbet et al. (2016b) study, ewes were housed throughout the study and fed hay and concentrate while being challenged with controlled numbers of *T. circumcincta* in a homologous challenge. In the study reported here ewes were raised outdoors on pasture where they faced continual heterologous parasite challenge, and were grazing rye grass white clover swards. This suggests that, in spite of a suitable level of humoral response, the functional protective element of vaccine-induced immunity was absent, or at least not apparent when using composite strongyle faecal egg counts from multiple parasite species as a measure of protection. It is perhaps notable that, in the Nisbet et al. (2016b) vaccine trial, pregnant ewes had faecal egg counts of zero at the time of V4 and only received a parasite challenge subsequent to V4, whereas in the work reported here ewes already had substantial faecal egg counts by the time of V4 in both years of the study. This suggests that these vaccines may have use prophylactically but not curatively.

Parasitic helminths produce an arsenal of immunomodulatory products which impact host immunocompetence (McNeilly and Nisbet, 2014) and can interfere with vaccine efficacy in helminth-infected hosts. For example, mice infected with the nematode *Litomosoides sigmodontis* had impaired vaccine-induced protection against both protozoan (*Plasmodium berghei*) and viral (influenza 2009 pH1N1) challenge pathogens compared with control mice with no *L. sigmodontis* infection (Kolbaum et al., 2012; Stetter et al., 2021). The presence of co-infecting GIN can substantially negatively impact immunity to other GIN species in natural-exposure situations (Vanalli et al., 2023) and we do not have any evidence that either prototype *T. circumcincta* vaccine has any efficacy against any of the co-infecting species present on the pastures in the experiment described here. Around the time of lambing, ewes also experience a relaxation in immunity, leading to the ability of GIN to establish in previously solidly-immune ewes (Gibson, 1973) and it is clear that this peri-parturient relaxation in immunity occurred in both years of the experiment. Overall, the immunosuppressive effects of co-infecting GIN, the absence of vaccine cross-protection against co-infecting GIN and the influence of the PPRI probably all contributed to the inability of either vaccine to protect against *T. circumcincta* infection in the work presented here. To address these issues, it may be necessary to design novel subunit vaccines which cross-protect against multiple GIN species and to alter the vaccination strategy to target multiple age cohorts of sheep over several seasons to reduce infection pressure.

## 3 Methods

### 3.1 Preparation of paddocks

In Year 1, a field that had previously been grazed by sheep was ploughed and re-seeded with grass seed, then divided into 12 adjacent paddocks (approximately 0.8ha) separated by fences. Ewes (n = 120) were infected with a dose of 15,000 L_3_ of mixed gastrointestinal parasitic nematode species common in the UK (60% *Teladorsagia circumcincta*, 9% *Trichostrongylus* sp., 4% *Chabertia ovina*, 27% *Oesophagostomum venulosum*). These ewes were placed in each paddock, 10 per paddock, throughout the grazing season, and rotated in their groups of 10 around paddocks every 2 weeks throughout spring and summer, with their spring-born lambs, to ensure uniform contamination of each paddock with parasites. Ewes and their lambs were removed from the paddocks in autumn of Year 2.

### 3.2 Vaccine administration to ewes

In spring of Year 3, 144 Scotch mule ewes, aged 3–5 years, and their lambs (2 per ewe) were treated with anthelmintic (Levacide, at the manufacturer’s recommended dose), placed onto these paddocks, 12 ewes per paddock, balanced for average weight, and rotated around the paddocks weekly. Lambs were removed from the ewes in late summer and, at that point until the end of the experiment in Year 5, no further rotation of animals was performed, i.e. 12 ewes were assigned to each set paddock from this point. The vaccination schedules from this point onwards, as well as the sample collections, are shown in **Figure 1**: In autumn of Year 3, the 72 ewes in six of the paddocks (“Vaccine” paddocks) were vaccinated, by subcutaneous injection, with a 2-antigen recombinant subunit *T. circumcincta* vaccine containing 50µg of each of mTci-APY-1 and Tci-MEP-1 plus 1mg of saponin adjuvant (VAX Saponin, Guiness Products) in a final volume of 1ml phosphate buffered saline (PBS) as described previously (Nisbet et al., 2019). Ewes were vaccinated on three occasions, two weeks apart during autumn and were given a fourth vaccination, as a booster, in the following spring (Year 4), six weeks prior to the estimated lambing date (Day 0, **Figure 1A**). Ewes in the six “Control” paddocks were unvaccinated throughout.

In autumn of Year 4, 26 of the older ewes were replaced in the experiment (14 from Control paddocks; 12 from Vaccine paddocks) with replacement 3-year-old ewes. All 72 ewes in the Vaccine paddocks were then vaccinated, by subcutaneous injection, with an 8-antigen recombinant sub-unit *T. circumcincta* vaccine containing 50µg of each of Tci-APY-1, Tci-MEP-1, Tci-CF-1, Tci-ASP-1, Tci-ES20, Tci-MIF-1, Tci-SAA-1 and Tci-TGH-2 plus 1mg of saponin adjuvant (VAX Saponin, Guiness Products) as described previously (Nisbet et al., 2016b). Ewes were vaccinated on three occasions, two weeks apart during autumn and were given a fourth vaccination, as a booster, in the following spring (Year 5), six weeks prior to the estimated lambing date (Day 0, **Figure 1B**). Ewes in the six “Control” paddocks were unvaccinated throughout.

### 3.3 Sampling during vaccine and challenge periods

Antigen-specific antibody levels (IgG and IgA) in serum were measured by ELISA as described in Nisbet et al. (2016b). Briefly, plates (Greiner Bio-one, high binding) were coated with each recombinant antigen (50 µl per well at a concentration of 1 µg/ml for IgG and 5 µg/ml for IgA). Serum from 18 ewes per treatment per timepoint (3 ewes from each paddock) was diluted 1:5000 for IgG and 1:10 for IgA in Tris Buffered Saline (20 mM Tris, 150 mM NaCl, pH 7.4) containing 0.1% Tween^®^ 20 (TBST). The secondary antibody used for IgG detection was mouse monoclonal anti goat/sheep IgG-HRP conjugate (Clone GT-34, Sigma A9452), used at 1:2000 and, for IgA, mouse monoclonal anti ovine/bovine IgA, used at 1:20,000 (Clone K84,2F9, Bio-rad AbD Serotec, MCA628GA). The tertiary antibody for detection of IgA was rabbit anti-mouse IgG-HRP conjugate (Dako P0260) used at 1:1000. Faecal egg count (FEC) analysis on samples acquired *per rectum* (Christie and Jackson, 1982) was performed at regular intervals throughout the study (**Figure 1**) as the principal measure of vaccine efficacy. The species composition of nematode egg samples was determined in samples derived from the ewes at lambing in each of the two years of the trial: Nematode eggs from each faecal sample were isolated using a modified salt flotation method and eggs collected were pooled by paddock. First-stage larvae (L_1_) were obtained using the larval culture method, as described in Melville et al. (2016). Each pool contained 59–100 and 383–500 L_1_s in Year 4 and Year 5 respectively and were stored in 75% ethanol at room temperature. Prior to DNA Extraction using DNeasy PowerSoil Pro Kit (QIAGEN, Aarhus, Denmark), ethanol was removed, and larvae bathed in 1x phosphate-buffered solution (PBS) (1/100 v/v) for 30 min to rehydrate. MT-PCR was completed using an automated diagnostic platform (Easy-Plex, AusDiagnostics Pty. Ltd., Beaconsfield, Australia): Primers were designed (AusDiagnostics Pty. Ltd., Beaconsfield, Australia) to amplify the internal regions of the ITS2 sequences of *Haemonchus* spp. (*H. contortus* and *H. placei*), *Teladorsagia circumcincta*, *Trichostrongylus* spp., *Oesophagostomum* spp. (*O. columbianum* and *O. venulosum*) and *Chabertia ovina*. Due to cross-reactivity issues, the ddASP gene was utilised for *Cooperia curticei* in place of the ITS2 region. The MT-PCR method, positive and negative controls and results analysis were performed as detailed in Roeber et al. (2017).

### 3.4 Statistical analyses

A generalised linear mixed model with negative binomial response and log link was fitted to the faecal egg count data to check for statistically significant differences between the control and vaccinated groups. The data from Years 3–4 and Years 4–5 were analysed separately. The data for the lambs and the ewes in Years 4 and 5 were also analysed separately. In all models, time and treatment were included as explanatory variables and random effects of paddock and ewe were included for the ewe models and random effects of paddock, ewe and lamb for the lamb models. Time was included in the model as a categorical variable. The interaction between the time and treatment was also considered, however this did not result in a robust model due to fitting issues and in absence of any suggestion of a treatment effect based on data visualisation and the fitting of simpler Poisson generalised linear mixed models and linear mixed models (to log(FEC+1)), the simpler model was chosen instead. All models were fitted in R 4.3.1 (R Core Team, 2013) using the lme4 library (Bates et al., 2015). The *p*-values were calculated using type II Wald chi-square tests from the Anova function in the car library (Fox and Weisberg, 2019). *p*-values less than 0.05 were considered statistically significant.

## Data availability statement

The raw data supporting the conclusions of this article will be made available by the authors, without undue reservation.

## Ethics statement

The animal study was approved by Moredun Research Institute Animal Welfare and Ethical Review Body (AWERB). The study was conducted in accordance with the local legislation and institutional requirements.

## Author contributions

AN: Writing – original draft, Writing – review & editing. TM: Writing – original draft, Writing – review & editing. DP: Writing – original draft, Writing – review & editing. YB: Writing – review & editing. MO: Writing – review & editing. DM: Writing – review & editing. LA: Writing – review & editing. GM: Writing – review & editing. RD: Writing – original draft, Writing – review & editing. SB: Writing – original draft, Writing – review & editing. FK: Writing – original draft, Writing – review & editing.
