# Supplementary figures and images for: Field testing of recombinant subunit vaccines against Teladorsagia circumcincta in lambing ewes demonstrates a lack of efficacy in the face of a multi-species parasite challenge
Source: Front Parasitol. 2024 Mar 25;3:1360029. doi: 10.3389/fpara.2024.1360029 (PMC11732088; doi:10.3389/fpara.2024.1360029)

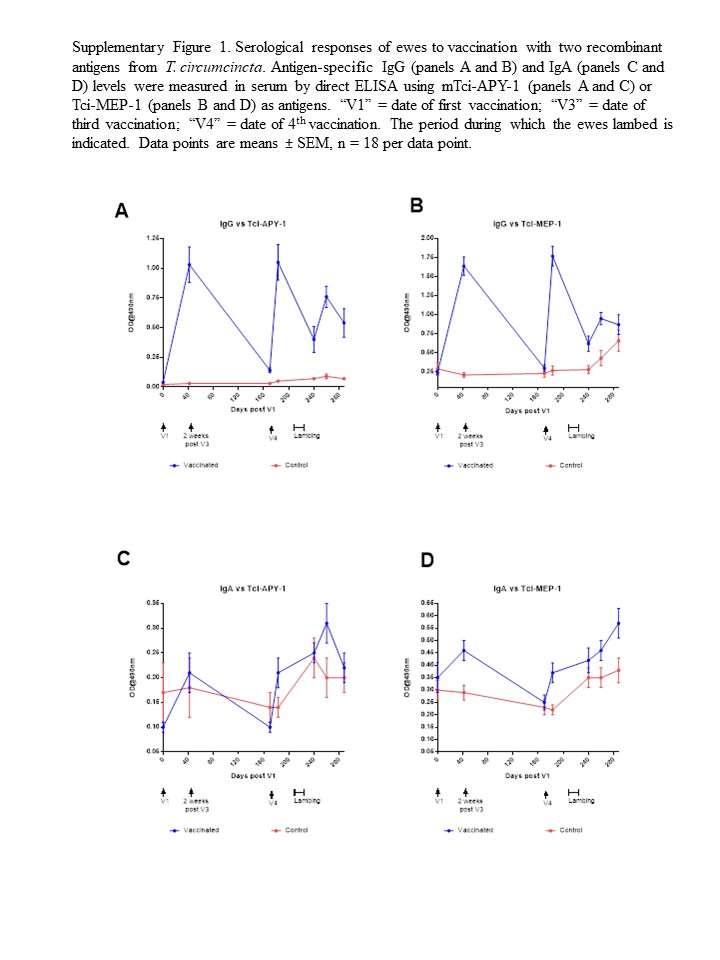

Supplement: Supplementary file 1 [file Image_1.jpeg]

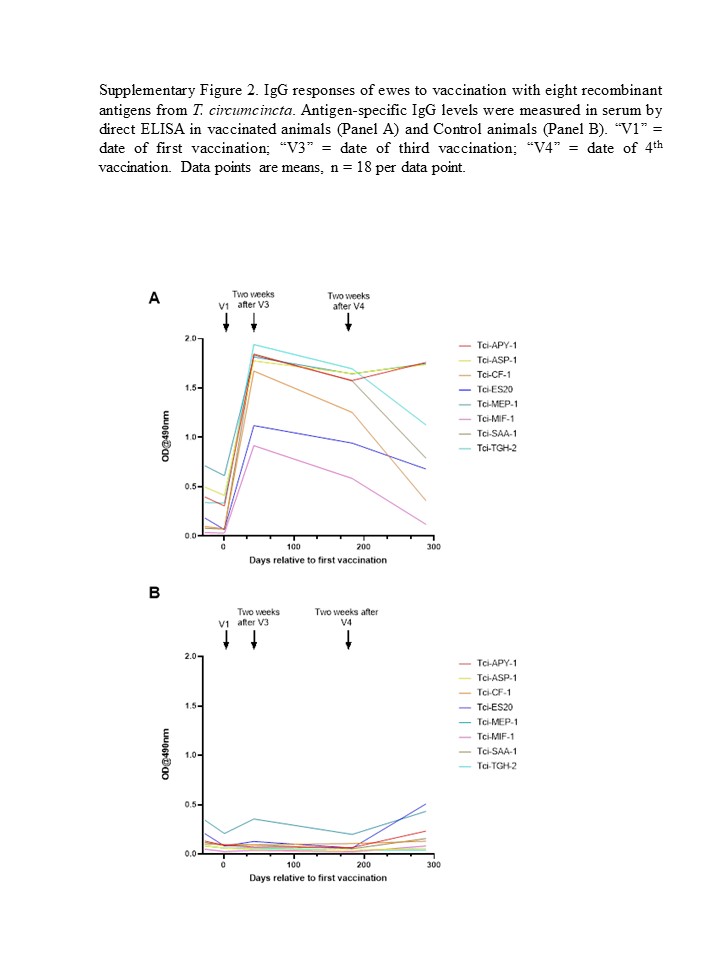

Supplement: Supplementary file 2 [file Image_2.jpeg]

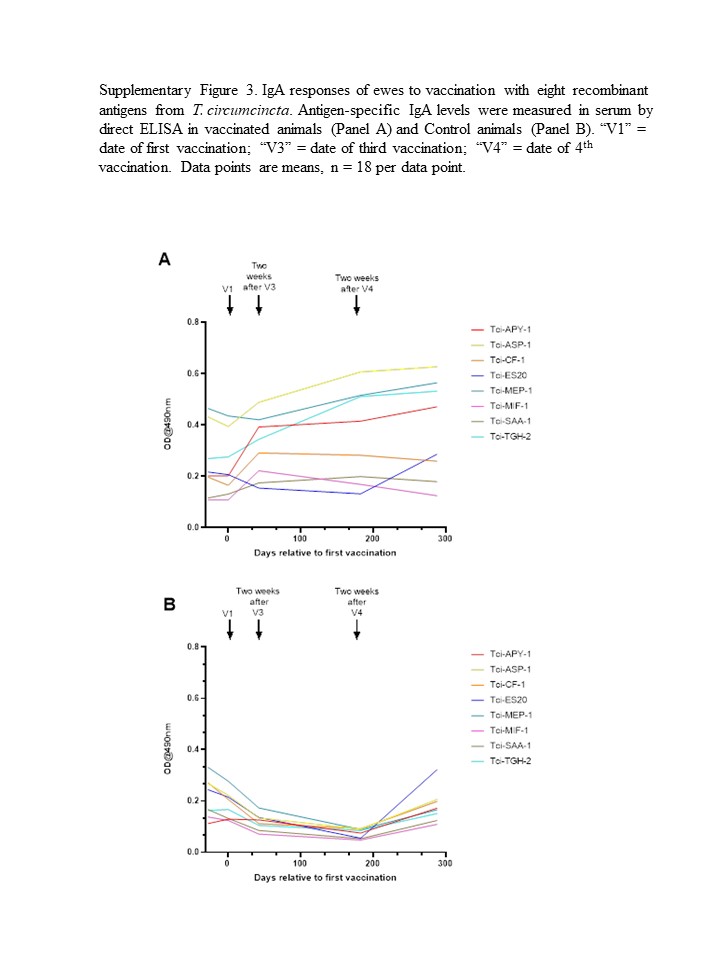

Supplement: Supplementary file 3 [file Image_3.jpeg]
